# Decreasing trends in patient satisfaction, accessibility and continuity of care in Finnish primary health care – a 14-year follow-up questionnaire study

**DOI:** 10.1186/1471-2296-15-98

**Published:** 2014-05-15

**Authors:** Risto Raivio, Juhani Jääskeläinen, Doris Holmberg-Marttila, Kari J Mattila

**Affiliations:** 1 Primary Care Unit, Joint Authority for Päijät-Häme Social and Health Care Group, Keskussairaalankatu 7, FI-15850 Lahti, Finland; 2 School of Medicine, Department of General Practice, FI-33014 University of Tampere, Tampere, Finland; 3 Centre of General Practice, Pirkanmaa Hospital District, P.O. Box 2000, FI-33521 Tampere, Finland

**Keywords:** Accessibility, Continuity, Patient satisfaction, Primary health care, Questionnaire survey

## Abstract

**Background:**

The aim here was to explore trends in patient satisfaction with primary health care and its accessibility and continuity, and to explore whether through reforms and improvements some of the essential goals had been achieved over a 14-year period of time in Finland.

**Methods:**

Nine questionnaire surveys were conducted over a period of 14 years among patients attending within one week in the 65 health centres in the Tampere University Hospital catchment area. A total of 147,394 responded out of a sample of 333,648 patients. The response rate varied yearly from 53% to 37%.

**Results:**

Patient satisfaction with care in Finnish health centres decreased by nearly 9 percentage units from 1998 to 2011. The fall-off was most marked in the age-group over 64 years. There was a 20 percentage unit’s reduction in ease of access as reported by patients. Respondents also reported that the continuity of care had deteriorated.

**Conclusions:**

Despite major reforms in Finnish health care policy, patients seem to be less satisfied. Our findings challenge both Finnish authorities and GPs to improve the accessibility and continuity of care in primary health services.

## Background

Accessibility and continuity of care are important aspects of good general practice and essential in efforts to improve the quality of performance [1-4]. Patients tend to attach particular importance to availability and accessibility, as also to the possibility of consulting the same GP [5,6]. There is also a positive correlation between patient satisfaction, continuity and accessibility of care, and medical treatment outcomes [7,8].

The Finnish health care services offer universal coverage over a comprehensive range of health needs implemented primarily by publically owned and operated organizations [9]. Primary health care service is provided mainly by municipal health centres. Recent decades have brought changes in Finnish health care guidance. The municipalities have sought to improve - or at least maintain - the quality of primary health care and increase its resources [Additional file 1]. Finnish National Health Programs [10,11] have emphasized a new standard of access to treatment and care. A National Health Care Guarantee introduced into the Finnish law in 2005 defined maximum waiting times for hospital and primary care services [12], and in 2007 the Government adopted a new reform programme known as the PARAS project to restructure local government and its services [13]. The latest national development programme for social and health care (the KASTE programme) emphasizes the significance of client feedback in organizing and producing health services and a new focus in primary care on the management of chronic diseases [14].

International studies have shown patients using primary care services to be generally fairly satisfied. There is nevertheless variation between different patient groups and primary health care organizations regarding the level of satisfaction with access to and continuity of care [15-17]. Prior to the present inquiry no systematic longitudinal studies have been undertaken measuring general patient satisfaction with access to and continuity of care in Finland.

The aim here was to ascertain how patients’ general satisfaction and the accessibility and continuity of care changed in Finland during the 14-year period covered (1998–2011). Our hypothesis was that during this period general patient satisfaction would have improved as a result of recent reforms and improvements in the Finnish health care system.

## Methods

The Department of General Practice at the University of Tampere sent a questionnaire to 65 primary health care centres in 1998, 1999, 2000, 2001, 2003, 2005, 2007, 2009 and 2011 [18]. The paper was distributed every study year among patients attending for treatment during one particular week. The questions were based on international studies [5] and adapted to the special characteristics of Finnish primary health care.

The questionnaire [Additional file 2] was piloted in the Pirkanmaa area in 1998, at which time 9 276 patients responded. In 1999, the inquiry was extended to primary health care centres located in the catchment area of Tampere University Hospital. There are 65 health centres in this area, the total population being 1.2 million. Only basic demographic data, such as gender and age of the participants, were collected.

Data were always collected during the same calendar week in September. The reception staff distributed the questionnaire to patients visiting physicians and nurses due to illness from Monday to Friday between 8 a.m. and 4 p.m.

Patients returned the anonymously filled questionnaires into a box in the waiting room after visiting the practice. The health centres collected the questionnaires and sent them to the Department of General Practice at the University of Tampere where the data was recorded.

We collected no data on non-participants or on patients not originally included in this study. In principal, any patients visiting any of the participating health centres due to an illness during the study week had an opportunity to voluntarily participate to the study.

Patient satisfaction was assessed with the statement: “The service in the health centre was so good that I can recommend it to my family and friends”. The response alternatives were: “I totally agree”, “I agree”, “I disagree” and “I totally disagree”. Since the objective in health service is complete satisfaction, we sought particularly to ascertain the proportion of respondents who gave the answer “I totally agree”, this being considered to stand for unreserved satisfaction with care.

Access to care was surveyed with the question: “How easy did you find it to call the health centre when making an appointment?” The respondents graded the service on a scale traditional in the Finnish school system and familiar to all patients. The grades ranged from 4 (“very poor”) to 10 (“excellent”). The highest mark 10 (Top-box) represented the best possible availability.

Continuity of care was assessed with the question: “When visiting the health centre, do you usually see the same doctor?” the alternatives being “yes” or “no”. The answer “yes” was taken to represent personal and longitudinal continuity of consultation with a specific doctor. In statistical analysis we used frequencies, percentages and cross-tabulation. The analyses were carried out with the SPSS predictive analytics software.

### Ethical approval

This study was based on the ethical principles prevailing under Finnish law. The research was non-invasive and not aimed at any individual person. The research data were based on information provided by respondents giving their opinions anonymously and voluntarily for research purposes. Neither the personal nor medical history of the respondents was used in the research, nor will the survey results be combined with any other data. Responses cannot be used to identify individual respondents. Health centres participated voluntarily in the research.

The concurrent joint Ethics Committee of the University of Tampere and Tampere University Central Hospital considered 6.8.2013 in their meeting that according to the Finnish legislation, no ethical assessment or approval was mandatory for our study.

## Results

Of the 65 primary health care centres in the area, 50 participated in the study at least six times and 63 at least four times. During the data collection periods, 333,648 patients visited the practices, and 147,394 responded. The response rate varied yearly from 53% to 37% [Table 1].

**Table 1:** Total number of patients visiting the health centres during week 39 between 8 a.m. and 4 p.m., participation of hospital districts, health centres, number of respondents and response rate by year

**Year**	**Hospital districts participating**	**Health centres participating**	**Patients**	**Respondents**	**Response rate**
	**N**	**N**	**N**	**N**	**%**
1998	1	22	19 399	9 276	47.8
1999	5	60	56 397	29 936	53.1
2000	5	64	52 517	24 163	46.0
2001	5	56	39 584	16 940	42.8
2002					
2003	5	49	36 917	15 036	40.7
2004					
2005	5	53	39 950	15 724	39.4
2006					
2007	5	61	36 630	15 988	43.6
2008					
2009	5	53	25 304	10 493	41.5
2010					
2011	5	32	26 950	9 838	36.6
Total	5	65	333 648	147 394	44.2

The median age of respondents was 51 years. The largest age-group was the 70-to-74-year-olds. In every age-group except for children, the majority of respondents were women. Two thirds of the respondents had been seen by a doctor, one third by a nurse. The characteristics of patients and respondents during the follow-up are presented in Table 2*.*

**Table 2:** The proportion (%) of gender, age, consultation with GP and nurse and urgency of consultation by study years

**Year**		**1998**	**1999**	**2000**	**2001**	**2003**	**2005**	**2007**	**2009**	**2011**	**Total**
Gender											
	Female	63.8	63.7	63.9	62.3	62.7	63.5	63.1	61.0	61.2	63.0
	Male	36.2	36.3	36.1	37.7	37.3	36.5	36.8	39.0	38.8	37.0
Age											
	<18	6.7	7.3	6.3	6.5	7.7	5.2	6.0	6.3	6.9	6.5
	18-64	62.6	61.9	61.7	61.8	59.1	61.5	59.2	57.0	57.8	60.6
	>65	30.7	30.8	32.0	31.7	33.3	33.3	34.7	36.7	35.4	32.9
Consultation with											
	GP	69.8	69.1	65.2	66.5	67.3	68.9	68.2	63.9	65.4	65.1
	Nurse	30.2	30.9	34.8	33.5	32.7	31.1	31.8	36.1	34.6	34.9
Urgency											
	Acute	28.3	25.7	27.2	28.7	29.2	28.5	30.3	28.7	31.6	28.3
	Subacute		18.1	19.6	19.1	20.8	21.8	21.8	18.3	19.3	23.1
	Not urgent		56.3	53.2	52.2	50.0	49.6	47.9	53.0	49.1	48.7

(Number of yearly respondents is presented in Table 1).

**Figure 1:**
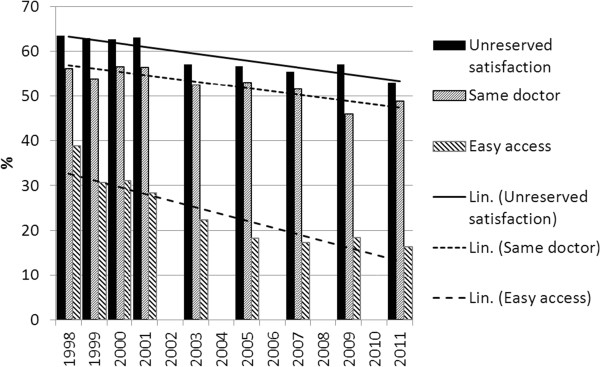
The proportion of patients expressing unreserved satisfaction, meeting the same doctors or same nurses and having easy access to health centre.

During the study period patients were well satisfied with health centre services. The proportion of those who agreed or totally agreed with the statement “The service in the health centre was so good that I can recommend it to my family and friends” was 97% in 1999 and 95% in 2011. The proportion of those who totally agreed with the statement was 59,8%. The main trend suggests a decrease in satisfaction [Figure 1]*.*

**Figure 2:**
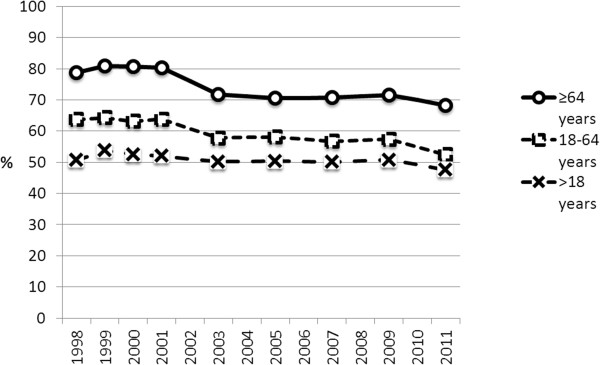
Proportion (%) of patients expressing unreserved satisfaction by age groups and years of survey.

Satisfaction with the services as a whole varied between health centres, the range being 39% to 95% in 1999 and 42% to 79% in 2011. Satisfaction also varied across age-groups [Figure 2]. The oldest group yielded the highest satisfaction rate, the youngest the lowest. The most marked change in satisfaction was among over 64-years-olds and appeared between study years 2001 and 2003.

Patients reporting complete accessibility during the years 1999 to 2011 decreased notably during that time [Figure 1], with only 16% of the participants reporting that in 2011, while the range between health centres varied from 14% to 61%. Accessibility was highest for the oldest age-group. However, the same group also showed the greatest decrease during the 14-year study period. The proportion of those patients who reported to being seen by the same doctor in a health centre during the whole study period was 53.3%.

Continuity of care had deteriorated [Figure 1]. The range between health centres was 13% to 98% in 2011. Regarding continuity of care there were no differences between age-groups.

## Discussion

The results of the present study show that people using primary health care services in Finnish health centres are generally fairly satisfied with them. Nevertheless, overall satisfaction diminished during the 14-year study period. Likewise fewer patients reported good access to and continuity of care.

The findings here provided no support for the hypothesis that the recent reforms and improvements in Finnish health care would have improved patients’ satisfaction with care on the whole – and especially with regard to access to and continuity of care.

This was the first longitudinal, systematic inquiry into patient satisfaction in primary health care in Finland. The strength of such a repeated sample was that all 65 centres in the district were invited to participate, and 76% participated in at least six out of eight rounds. During the fourteen-year study period we were able to gather an extensive sample of patient opinions. In this study area of 1.2 million inhabitants there are small rural health centres and large health centres in the conurbations. According to the official Finnish health and statistical registers, our study area seems to represent the rest of the country quite well in terms of level and time trends in volume and costs of primary health care as a whole and the health centre visits in particular. As the patients who chose to answer our inquiry were those who had an opinion and wanted to express it, the respondents’ views cannot be taken to represent those of the whole population of Finland but presumably those of the section using health care services in health centres.

The idea for our questionnaire came originally from European patient satisfaction studies [5] and was applied to the Finnish health care system and validated in our pilot study in 1998. We were aware of the challenges involved in studies using a questionnaire survey [19]. Reliance on personnel to distribute questionnaires is flawed as a mechanism and involves some kind of sampling bias. This notwithstanding, the same flaws apply to the data throughout, and it is thus reasonable to conclude that the comparison over time remains robust. The low overall response rate (45%) was a limitation of this study. Nevertheless, we consider that our data and the process of assessing patients’ views were feasible and comprehensive [18]. The comparability of different study years was ensured by implementing the questionnaire identically every study year.

A number of factors influence patient satisfaction [15-17,20-22]. They may be related to patient characteristics or to features of the health care system. On the other hand, patient satisfaction alone is not a guarantee of good and efficient health care. Good communication during consultation increases satisfaction [23]. This aspect was not included nor analysed in our study.

Many studies have shown that patients’ age, gender, perceived health status at the time and socioeconomic status have an effect on their satisfaction with health care services. According to the official health registers the variables mentioned did not change significantly in Finland over the period covered. The most marked decrease in patient satisfaction noted here was in the group over 64, who tend to have multiple diseases in long-term conditions – and also an increased need of health services.

Patients in primary care appreciate ease of access and continuity of care [2,7,8,15,20]. Ratings of the access to the health services were conspicuously low, eminently in the last study year 2011 and among the elderly. Also ratings of the continuity of care had declined by 2011 and the variation between the health centres in satisfaction with the accessibility and continuity of care was significantly high. Elderly patients with chronic diseases attach particularly great importance to the continuity of care and a long treatment relationship with a particular doctor.

Finnish health care centres underwent a number of changes during the study years [24,25]. This may partly explain the falling trend in patient satisfaction with the services in general and with the accessibility and continuity of care in particular. Some of the changes in question were influenced by societal change, financial austerity and a lack of experienced primary care professionals.

In the 1990s the Ministry of Education radically reduced student intakes in the medical schools, since when the shortage of family doctors and GPs in primary health care has become more acute. This deficit – also associated with demanding working conditions, heavy workload and ageing and demanding patients – has led to smaller numbers of GPs working in primary health care. The consequent poor accessibility and continuity of care has led to a decrease in patient satisfaction. At the same time young doctors have preferred a career in hospitals and in occupational or private health care instead of working in health centres.

During the study period, occupational healthcare was growing strongly, and the municipal health centres were struggling to get their share of the total health care resources. In ten years since 2000, the absolute number of non-urgent physician visits in health centres went down by 20% nationally. This, in turn, has led to a substantial increase in waiting times for non-urgent care [26]. Between 2000 and 2010, there was a net increase of about 25% in the total number of active physicians in Finland. However, this growth was directed almost totally to other parts of Finnish health care, not to the municipal health centers [26].

Legislation on maximum waiting times has existed in Finland since 2005. There is, however, no indication so far that the new modes of procedure have had a positive effect on the accessibility of primary health care. While the legislation sets the framework for how and when certain medical conditions ought to be diagnosed and treated, it may also exacerbate inequality of care. The legislation on maximum waiting times raises expectations in the general public which may not always be met since it only covers specified medical conditions. The proportion of non-urgent visits has decreased over the years. Regarding resource allocation, the legislation may result in more resources being allocated to emergency outpatient clinics and specialized care. At the same time care for patients with chronic illnesses and complicated psychosocial problems may receive even less resources than before. The poor access to health centres has proved a marked drawback, and recent efforts have focused on developing a call-back technology to ease access to services.

The implemented and planned national health care programmes and legislation in Finland were designed to improve the health services. The general strategic reforms with weak implementation methods might not have been able to resist the strong pressures within the service provision system.

For health care policy to be successful the system should focus on providing more people with better accessibility and personally focused, humanely conceived comprehensive and coordinated primary care [27]. Policy-makers and health care professionals should collaborate in efforts to narrow the gap between public expectations and patients’ experience [28]. One of the latest development programmes is a new way to organize long-term care and freedom of choice in the health care services. The chronic care model [29,30] and freedom of choice for the population [31] draw patients, professionals and decision-makers together in improving the quality, fairness and effectiveness of and satisfaction with care, and may help to focus on those patients who are most in need of care.

## Conclusions

It would appear that despite several programmes and acts to improve health care services in Finland during the last few decades, neither patient satisfaction nor the accessibility and continuity of care has improved in primary health care. The challenge is to achieve a better understanding of the factors underlying the declining trend and causing differences in patient satisfaction between the various health centres. In any case the findings here indicate that action is called for to improve the continuity and accessibility of care.

## Competing interests

The authors declare that they have no conflict of interests.

## Authors’ contributions

RR, JJ, DH-M and KJM designed the study and participated in the acquisition of data. RR and KJM analysed and interpreted the data. RR, JJ, DH-M and KJM drafted the manuscript, critically revised the article and have read and approved the final manuscript.

## Pre-publication history

The pre-publication history for this paper can be accessed here:

http://www.biomedcentral.com/1471-2296/15/98/prepub

## Supplementary Material

Additional file 1 The list and timeframe of health policies and acts mentioned in the article. Click here for file

Additional file 2 Patient Survey (questionnaire). Click here for file
