# Supplementary material for: Decreasing trends in patient satisfaction, accessibility and continuity of care in Finnish primary health care – a 14-year follow-up questionnaire study
Source: BMC Fam Pract. 2014 May 15;15:98. doi: 10.1186/1471-2296-15-98 (PMC4030039; doi:10.1186/1471-2296-15-98)
Supplement: Additional file 1 — The list and timeframe of health policies and acts mentioned in the article. [file 1471-2296-15-98-S1.doc]

**The list and timeframe of health policies and acts mentioned in the article**

**From 2001 to 2015.** [**Government resolution on the Health 2015 - Public Health Programme.**](http://pre20031103.stm.fi/english/eho/publicat/ehocontents17.htm) (Publications of the Ministry of Social Affairs and Health 2001:6). [<http://pre20031103.stm.fi/english/eho/publicat/ehocontents17>]

“The Government Resolution on the Health 2015 public health programme outlines the targets for Finland’s national health policy **for the next fifteen years.** The main focus of the strategy is on health promotion, not so much on developing the health service system. The foundation for the strategy is provided by the Health for All programme of the WHO, which was revised in 1998. The strategy is a continuation of the Finnish national HFA 2000 programme.”

**From 2002 to 2008.** [**Decision in Principle by the Council of State on securing the future of health care.**](http://www.stm.fi/english/eho/publicat/ehocontents68.htm) (Brochures of the Ministry of Social Affairs and Health 2002:6). [http://pre20031103.stm.fi/english/eho/publicat/bro02_6/chap1.htm]

“The principle of access to treatment within a reasonable period will be embodied in legislation by the year 2005. The Ministry of Social Affairs and Health will more specifically guide access to non-urgent treatment through decrees and directives that will come into force **on January 1st 2005**.”

**From March 1^st^ 2005.** [**Health care services are improving. Timeframes for access to non-emergency treatment.**](http://www.stm.fi/Resource.phx/publishing/documents/3524/index.htx) (Brochures of the Ministry of Social Affairs and Health 2004:13). [http://www.stm.fi/en/publications/publication/_julkaisu/1057139#en]:

”From the beginning of March 2005, non-urgent examination and treatment at health centres and hospitals must be provided within clear timeframes.”

**From 23.02.2007 to 31.12.2012.**  Kunta- ja palvelurakenneuudistus (PARAS). **Municipal and Service Reform to Assure Equal Social and Health Services for All Residents.** Ministry of Social Affairs and Health, Finland. (Text available only in Finnish)

[http://www.stm.fi/vireilla/kehittamisohjelmat_ja_hankkeet/paras]

“The project to restructure Finnish local government and services aims at ensuring equal social and health services for all residents. Prerequisites for this development are intact and functioning municipal structures, strengthening of the local service structures and reorganization of services on a broader population base, inter-municipal cooperation, and new ways of working. The reform process is based on framework legislation, which came into force in 2007, the Act on the Restructuring of Local Government and Services (169/2007).”

**From 2008 to 2011. Health and Wellbeing: our Common Goal.** National Development Plan for Social and Health Care Services. Kaste Programme 2008–2011. (Brochures of the Ministry of Social Affairs and Health 2008:5). [[http://www.stm.fi/en/publications/publication/_julkaisu/1068257#en](http://www.stm.fi/en/publications/publication/_julkaisu/1068257#en)]

“The Kaste programme is the national development plan for social and health services, which covers the period 2008-2011. The main aims of the programme are based on the long-term strategic objectives for the social and health administrative sector. The aims are that: municipal inhabitants' social inclusion will increase and levels of social exclusion will be reduced, the municipal inhabitants' wellbeing and health will increase, inequalities in wellbeing and health diminish, and the quality, effectiveness and availability of services for the municipal inhabitants will improve and regional inequalities will be reduced. The aim is to achieve the objectives of the programme by prevention and early intervention by ensuring the sufficiency of staff and strengthening skills, and by social and health care services functioning as an integral whole and using effective models of operation.”

**From May 1^st^ 2011. The New Health Care Act. The Memorandum of the Working Group Preparing the Health Care Act.** Report 2008:28. Text in Finnish, summary in English. [http://www.stm.fi/julkaisut/selvityksia-sarja/nayta/_julkaisu/1066999#fi]

**Health Care Act improves the status of clients.** Press release 194/21010. Ministry of Social and Health Affairs. [[http://www.stm.fi/en/pressreleases/pressrelease/view/1512997#en](http://www.stm.fi/en/pressreleases/pressrelease/view/1512997#en)]

“The new Health Care Act aims at improving the status of clients as well as the quality of services and care. This is to be achieved by giving the clients freedom to choose the place of care, by guaranteeing equal access to services and by improving the quality of care and patient safety.

The Government decided on the contents of the bill in June 2010. The new Health Care Act entered into force on 1 May 2011.”

**From 2008 to 2011. National Action Plan to Reduce Health Inequalities 2008-2011.**
Ministry of Social Affairs and Health's publications 2008:25. [http://pre20090115.stm.fi/pr1227003636140/passthru.pdf]

“The objective of the Action Plan is to reduce social inequalities in work ability and functional capacity, self-rated health, morbidity and mortality by leveling up. Narrowing of the inequalities will have a positive effect on public health and help to secure the services as the population ages, raise the employment rate and restrain the costs.

The National Action Plan to Reduce Health Inequalities is closely linked with the Government’s Health Promotion Policy Programme. The Action Plan will also for its part implement the aim of the national “Health 2015" programme to reduce mortality differences by a fifth by 2015.”

**From 2009 to 2011. The Functioning Health Centre – Action Programme 2008.** Brochures of the Ministry of Social Affairs and Health 2008. Text available only in Finnish. [<http://www.stm.fi/vireilla/kehittamisohjelmat_ja_hankkeet/toimivaterveyskeskus>]

“The Functioning Health Centre Programme is designed to strengthen the Finnish primary health care system by developing health care practices, administration and management as well as health care education and research. The programme focuses in particular on the availability and sufficiency of the medical staff, which are key factors in the functioning of health care centres. The reforms included in the programme will be gradually introduced in health care centres in 2009 –2011.”
