# Supplementary material for: Decreasing trends in patient satisfaction, accessibility and continuity of care in Finnish primary health care – a 14-year follow-up questionnaire study
Source: BMC Fam Pract. 2014 May 15;15:98. doi: 10.1186/1471-2296-15-98 (PMC4030039; doi:10.1186/1471-2296-15-98)
Supplement: Additional file 2 — Patient Survey (questionnaire). [file 1471-2296-15-98-S2.doc]

Health centre .................................................................. Community/ Area code ...............

Surgery /clinic ......................................................................... Surgery /Clinic code ...............

Day of week seen  Monday  Tuesday  Wednesday  Thursday  Friday

INSTRUCTIONS:

The aim of this survey is to improve the services provided by the health centre. **Please evaluate the health centre’s services based on your visit today**. Please select only one answer for each question. In evaluating the service, please select the (Finnish) school grade that best corresponds to your opinion (numbers 4-10, 4 being the lowest and 10 the highest grade). If you haven’t needed a particular service or don’t have an opinion on it, please select the appropriate option from the ones preceding the grades.

BACKGROUND INFORMATION (only for statistical purposes)

**Are you**  female **or**  male? **How old are you?** ...... years old

**What was the reason for your visit?**  a/e related  other urgent matter  non-urgent

**Do you have a particular doctor appointed for you at the health centre?**  yes  no

**In the last 12 months, how many times have you visited the health centre prior to this visit?**

Physician’s surgery ....... times

Community health nurse’s /nurse’s appointment ....... times

**In the last 12 months, have you needed to visit the a&e in the evenings, nights or during the weekend?**

 yes  no

**When you visit the health centre, do you usually see the same**

doctor?  yes  no

community health nurse /nurse?  yes  no

**How did you book your appointment for today?**

 By phone  In person  I didn’t book an appointment before coming

**Which health care provider did you see today?**

Please select only the health care provider you saw today and whose service you are evaluating with this questionnaire.

 doctor

 community health nurse/nurse

## THE SURVEY CONTINUES ON THE OTHER SIDE OF THE FORM

I haven’t needed the service

Don’t know/ can’t say

# Booking an appointment

How easy did you find it to get through on the

phone to the health centre?   4 5 6 7 8 9 10

How would you rate the receptionist’s behaviour when you

were booking the appointment (respect, geniality and politeness)   4 5 6 7 8 9 10

Did you get to see the doctor or nurse you wanted to?  yes  no

# Registration

# How smooth was the registration?   4 5 6 7 8 9 10

Did you get to see the doctor/ nurse at the agreed hour?   4 5 6 7 8 9 10

# Evaluating the surgery

How did the person you saw behave during the surgery?   4 5 6 7 8 9 10

(respect, geniality and politeness)

Did you get enough information about tests and their results?   4 5 6 7 8 9 10

Did you get the information you needed about your medicines

and their effects?   4 5 6 7 8 9 10

Did you get information about the treatment options

for your particular health problem?   4 5 6 7 8 9 10

Did you get clear and adequate instructions for further   4 5 6 7 8 9 10

care and treatment?

Did the doctor/nurse listen to your problems and did s/he show

interest toward you and willingness to answer your questions?   4 5 6 7 8 9 10

# Your opinion about the visit to the health centre

Did you feel that your matters were dealt with confidentiality?   4 5 6 7 8 9 10

Did you get help for your health problem?   4 5 6 7 8 9 10

Was enough time provided for dealing with your matters?   4 5 6 7 8 9 10

# The service at the health centre was so good that I am able to recommend it to my family and friends.

 I agree

 I agree to some extent

 I disagree to some extent

 I disagree

PLEASE MAKE SURE YOU HAVE ANSWERED ALL THE QUESTIONS THANK YOU
